# Supplementary material for: Household indebtedness and well-being: Evidence from Australia
Source: PLoS One. 2025 Dec 17;20(12):e0337006. doi: 10.1371/journal.pone.0337006 (PMC12757963; doi:10.1371/journal.pone.0337006)
Supplement: Attachment — Submitted filename: Response Letter.pdf [file pone.0337006.s001.pdf]

Point by point response letter

Dear Editors,

The authors thank the Academic Editor and both reviewers for the thoughtful and constructive assessment. The manuscript titled “Household Indebtedness and Well-being: Evidence from Australia” has been revised carefully to address every point raised in the decision letter and the referee reports; the changes are itemised below and the revised manuscript reflects these modifications throughout. The revised Tables 4, 5, and 6 already implement the requested presentational and interpretive updates: coefficients for debt to income ratios are now expressed per ten percentage point increase, ninety five percent confidence intervals are included, pooled logit results for obesity are presented as average marginal effects in percentage points, and a standardised analysis is reported for the continuous outcomes. The text has been amended to make these estimands explicit; Methods document the rescaling and the presentation of average marginal effects; Results and Discussion have been edited to remove language that implied effects for a one hundred percentage point swing.

Response to Academic Editor

Comment 0.1

Add model fit information so that readers can gauge how much variation the models explain and how the key regressors contribute to fit.

Response 0.1

Tables 4–6 now report model results as follows: pooled OLS reports R^2; fixed-effects models report within R^2; and the pooled logit model for obesity reports average marginal effects (AMEs). The Methods clarify these choices and note that these quantities are not comparable across model families; table notes repeat this guidance for transparency.

Comment 0.2

Ensure that the submission conforms to PLOS style requirements including file naming and required sections.

Response 0.2

The main text has been formatted to match the PLOS ONE style using PLOS ONE LaTeX template.

Comment 0.3

Align the Funding Information and Financial Disclosure statements; the grant numbers do not currently match.

Response 0.3

The two sections now contain identical funder names and grant identifiers; the text also restates that funders had no role in study design, analysis, interpretation, writing, or the decision to submit.

Comment 0.4

Revise the Data Availability statement; the repository originally cited is not acceptable. Deposit the minimal data set in a stable public repository and provide a persistent identifier.

Response 0.4

This study uses confidential unit-record data from the HILDA Survey, which are available only to approved users via application to the data custodian; the authors are not permitted to redistribute the unit-record data.

Comment 0.5

Review the reference list for completeness and accuracy; remove or annotate any retracted items; evaluate any works suggested by the reviewers.

Response 0.5

The reference list has been checked against current records to the best of our knowledge as of October 2025; no cited item appears as retracted. The manuscript now incorporates reviewer suggested clarifications to the background and definitions without adding non peer reviewed sources.

Comment 0.6

Preflight figure files through the PACE diagnostic tool.

Response 0.6

N/A

Response to Reviewer 1

Comment 1.1

Rescale the effects so that results are stated per ten percentage point change, given that the credit card debt to income ratio is trimmed below one and the current prose implies effects from zero to one hundred percent. Consider effect sizes in standard deviation units for comparability across outcomes.

Response 1.1

The debt to income ratios for credit card and mortgage balances were divided by ten prior to estimation; all coefficients and average marginal effects now correspond to a ten

percentage point increase. Methods state this respecification; Results and the Abstract have been edited to ensure consistent interpretation. The revised Table 4 also includes a standardised panel in which health satisfaction and the Mental Component Score are transformed to z scores; the text reports these standard deviation units where appropriate.

Comment 1.2

For the pooled obesity model, the coefficients reported previously were on the log odds scale. Report average marginal effects with ninety five percent confidence intervals, preferably per ten percentage point change.

Response 1.2

The pooled obesity specification now reports average marginal effects in percentage points per ten percentage point increase in the debt to income ratio; 95% confidence intervals appear in the tables. The table notes and the Methods section specify the estimand and the presentation.

Comment 1.3

Avoid exposure misclassification in the mortgage stress models; either restrict to mortgage holders or model interactions with a mortgage holder indicator; clarify this choice in the table note.

Response 1.3

All mortgage stress models are already restricted to respondents with an active mortgage as mentioned (Could not pay the mortgage); the Methods section states this analytic population and Table 5 notes the sample restriction explicitly. The Results subsection on mortgage stress opens with a sentence that reiterates this restriction.

Comment 1.4

Temper the robustness claims when relaxing the mortgage trimming threshold; higher caps strengthen credit card associations for mental health while the association with health satisfaction weakens.

Response 1.4

The robustness subsection has been revised to adopt the suggested nuance. The text now states that fixed effects associations are most stable for the Mental Component Score, with weaker and less stable patterns for health satisfaction once mortgage trimming is relaxed; the always employed subsample yields consistent patterns for mental health.

Comment 1.5

Clarify the confidence interval and unit conventions in the tables.

Response 1.5

All regression tables include harmonised notes that state the unit of the coefficients, that parentheses contain standard errors, and that brackets contain ninety five percent confidence intervals. These notes also document the rescaling by ten percentage points and the use of average marginal effects for the pooled obesity models.

Response to Reviewer 2

Comment 2.1

Define the term “stock measures” at first use in the background section.

Response 2.1

The Introduction now defines stock measures at first use as outstanding balances recorded at the interview date that capture the level of liabilities rather than flows such as missed repayments; this clarifies the conceptual distinction that motivates the empirical strategy.

Comment 2.2

Repair the sentence that begins “Building on [17]” by naming the author or authors.

Response 2.2

The sentence now reads: “Building on Keese and Schmitz, who used German panel data to demonstrate that repayment difficulties across consumer credit and mortgage liabilities undermine health, this study examines stock measures of debt in an Australian context.”

Comment 2.3

Strengthen table titles and notes so that a reader can understand units and notation without referring back to the text.

Response 2.3

Table now identify the unit of change for the regressors, the model family, and whether outcomes are standardised; table notes specify that parentheses contain standard errors and brackets contain ninety five percent confidence intervals, that coefficients for debt to income ratios represent changes per ten percentage point increase, and that pooled logit results for obesity are presented as average marginal effects in percentage points; fit measures are reported as described above.

Summary of key manuscript edits implemented

Comment 2.4

List the main changes introduced across sections for ease of verification.

Response 2.4

Abstract: removed language implying a one hundred percentage point swing and restated effects per ten percentage points; referenced confidence intervals in the tables. Methods: added a sentence in the Debt Measures subsection documenting division of ratios by ten; added a sentence in the Outcomes subsection that the standardised specifications convert the two continuous outcomes to z scores; added a paragraph in the Empirical Strategy subsection explaining that pooled obesity results are presented as average marginal effects with ninety five percent confidence intervals and that fit diagnostics are reported with guidance on interpretation; stated explicitly that mortgage stress analyses are restricted to mortgage holders. Results: edited Section 4.2 to state effects per ten percentage points and to report the standardised coefficients in Table 4 Panel B for context; edited Section 4.3 to begin with the mortgage holder restriction; edited robustness language to reflect stronger consistency for mental health. Discussion: acknowledged the standardised magnitudes and the robustness pattern. Tables 4 to 6: added fit measures; harmonised titles and notes; expressed all units per ten percentage points; presented average marginal effects for pooled obesity; included the standardised panel in Table 4.

The authors trust that these revisions address the Academic Editor's request and the reviewers' concerns in full. The manuscript now states the estimands clearly, documents the rescaling and presentation choices in Methods, reports fit diagnostics to aid interpretation, clarifies the analytic population for mortgage stress, strengthens the interpretability of tables, and resolves the definitional and citation issues in the Introduction.

Sincerely,

Corresponding Author
On behalf of all authors
